# A Data Mining Approach Identified Salivary Biomarkers That Discriminate between Two Obesity Measures

**DOI:** 10.1155/2019/9570218

**Published:** 2019-05-19

**Authors:** Ping Shi, J. Max Goodson

**Affiliations:** Department of Applied Oral Sciences, The Forsyth Institute, Cambridge, MA 02142, USA

## Abstract

**Background:**

A key mechanism of obesity involves dysregulation of metabolic and inflammatory markers. This study aimed to identify salivary biomarkers and other factors associated with obesity using an ensemble data mining approach.

**Methods:**

For a random cohort of over 700 subjects from 8137 Kuwait children (10.00 ± 0.67 years), four data mining methods were applied to identify important variables associated with obesity, including logistic regression by lasso regularization (Lasso), multivariate adaptive regression spline (MARS), random forests (RF), and boosting classification trees (BT). Each algorithm generated a variable importance rank list, based on an internal cross-validation procedure. An aggregated importance ranking was constructed by averaging the rank ordering of variables from individual list, weighted by the classification performance of respective models. Subsequently, the subset of top-ranking variables that were identified with at least three algorithms was evaluated by classification performance using receiver operating characteristic (ROC) analysis with bootstrap percentile resampling.

**Results:**

Obesity was defined either by the waist circumference (OBW) or by the body mass index (BMI) (OBWHO). We identified C-reactive protein (CRP), insulin, leptin, adiponectin, as salivary biomarkers associated with OBW, plus a clinical feature fitness level. A similar set of biomarkers was identified for OBWHO, but not including leptin. Tree-based clustering analysis revealed patterns that were significantly different between the OBW and OBWHO subjects.

**Conclusion:**

A data mining approach based on multiple algorithms is useful for identifying factors associated with phenotypes, especially in cases where relationships are not salient, and a consensus from multiple methods can help produce a more generalizable subset of features. In this case, we have demonstrated that evaluation using the waist circumference includes association with high levels of salivary leptin, which is not seen with evaluation by BMI.

## 1. Introduction

The Kuwait children's study is a large-scale study aimed at evaluating the etiology of obesity and development of metabolic syndrome in over 8,000 Kuwait children [1, 2]. It has a massive data collection, including anthropometric and clinical features, dietary survey, and molecular profiling of salivary protein biomarkers, metabolites, as well as microbes. Like other collection of complex datasets with hundreds of variables in large-scale cohort studies, it becomes a challenge to find associations between covariates and phenotypes [3]. The parametric modeling approach based on preformulated hypothesis is limiting, as it is unable to handle a large number of covariates simultaneously and search efficiently for unanticipated associations.

An alternative approach to the conventional hypothesis-driven analysis is data mining, which is a data-driven process to discover novel relationships in large quantities of data without any *a priori* hypothesis [4]. Data mining algorithms are nonparametric, rendering their applicability to various types of data, whose different characteristics can be tuned to by different algorithms. They are able to deal with large number of variables, and sometimes detect not only covariates with a strong main effect but also those with significant interaction effects but minimal main effects, which may not be possible in a conventional model. Usually, they can handle complex relationships between covariates and the outcome, accounting for nonlinear association in various ways. Furthermore, a salient feature in this type of approach is cross-validation procedure, which addresses the issue of generalization of models across samples, and avoids overfitting, a common problem in parametric modeling.

The present study focused on the salivary protein biomarker dataset, which was a randomly selected cohort derived from the entire dataset [1]. Development of obesity in children increases the risk of developing cardiovascular disease (CVD), type-2 diabetes, and other chronic diseases in later life, which, to a large extent, are mediated by adipokines and cytokines released from the adipose tissue [5, 6]. Therefore, biomarker studies enable us to gain insights into the etiology of obesity-related diseases, especially pathways leading to various pathologies. Some previous studies examined the relationship between these factors and obesity-related conditions in adolescents, mainly by association studies with hypothesis-based modeling using prespecified variables [7]. Our study employed a data-driven approach to identify crucial salivary biomarkers associated with obesity. We applied four algorithms to our dataset: logistic regression by lasso regularization (Lasso) [8], multivariate adaptive regression spline (MARS) [9], random forests (RF) [10], and boosting classification trees (BT) [11].

## 2. Materials and Methods

### 2.1. Data Source

The dataset was a random cohort of 744 subjects selected from the entire population (*n* = 8137) of Kuwait children's study, which had all the anthropometric and clinical measures as well as saliva samples collected between October 2, 2011 and May 15, 2012 [1, 2]. Biomarker assays were performed on the saliva samples from the random cohort using a multiplex bead platform (Luminex® 200, Austin, TX). The measured salivary biomarkers included insulin, C-reactive protein (CRP), adiponectin, leptin, IL-1*β*, IL-4, IL-6, IL-8, IL-10, IL-12P70, IL-13, IL-17A, resistin, MMP_9, MPO, MCP-1, TNF-*α*, VEGF-A, IFN-*γ*, and ghrelin, of which IL-17A, IFN-*γ*, and ghrelin were not included in the analysis due to a significant portion of missing values. Additionally, 18 samples with extreme values in measurements were excluded from subsequent analysis, based on their undue influence in an initial regression model. The values of biomarkers were standardized prior to analysis. Fitness was measured by heart rate elevation following a standard exercise [1] and then binarized using the median value from the original entire study population.

Both outcome measures for obesity were transformed to binary measures. Obesity was defined as having BMI (OBWHO) or waist circumference (OBW) in the 95^th^ percentile or higher within one's age and gender group [12].

### 2.2. Data Mining Algorithms for Variable Selection

Analysis of variable importance by lasso logistic regression (Lasso), multivariate adaptive regression spline (MARS), random forest (RF), and boosting classification trees (BT) was conducted using Salford Predictive Modeler v7.0 [13]. To obtain the final model for variable selection, an internal 10-fold cross-validation procedure was employed in all algorithms, except for random forest, which estimated its prediction error in the out-of-bag samples. Importance of each variable was measured according to different schemes in the respective method, which are defined as follows. In lasso, the importance measure was represented by the *β*-coefficients of the resulting logistic regression model. In MARS, as each variable was added to the model, reduction of the general cross-validation (GCV) statistics was used as the importance measure. In RF, the classification error rates on the out-of-bag sample were recorded for individual trees before and after permutation of a given variable, and the importance measure was based on the difference between the two error rates averaged over all trees. In BT, variable importance of a given variable was computed as the cumulative sum of improvement in node purity from all splits, across all trees up to a specific model size. Furthermore, based on the importance measure for each algorithm, the relative importance score was derived, which was expressed by rescaling the importance so that the most important variable on the top was denoted 100, and other variables were scaled by their values relative to that of the top one. In our study, we used a cutoff value of 0.2 to determine whether a variable was identified as an important factor.

### 2.3. Aggregation of Variable Rank List

To obtain a consensus from these four algorithms, aggregated rank ordering was created by the weighted average of individual ranking of each variable, with classification performance of the model that generated its ranking as the weight factor. Thus, for variable *j*, its aggregated rank *R*_*j*_^*f*^ is expressed as *R*_*j*_^*f*^=∑_*i*=1_^*n*^*ω*(*R*_*i*_), where *i* denotes the model, *ω* the weight of model *i* as defined by AUC, and *R*_*i*_ its rank in model *i*. Meanwhile, if one variable was selected by at least three algorithms, it was considered as the winner of the majority vote.

### 2.4. Evaluation of Classification Performance and Clustering Analysis

To evaluate the classification performance of the subsets of top-ranking variables in the aggregated rank ordering, ROC analysis was conducted using the biomarker analysis function of an online comprehensive tool suite MetaboAnalyst [14]. A 95% confidence interval was obtained for the ROC curve from bootstrap percentile resampling [15].

A clustering analysis was carried out based on the internal distance measures in random forest, available from Salford Predictive Modeler v7.0 [13]. This measure of proximity is the fraction of available trees, in which a pair of subjects landed on the same terminal node, out of the total number of trees. A multidimensional scaling (MDS) processing of the full proximity matrix generated a MDS display of the distance between all data points, which provided evidence of clustering.

## 3. Results

### 3.1. Identification of Factors Associated with Obesity as Defined by Waist Circumference or BMI

The cutoff value of 0.20 was used for selection of important factors from the variable importance list. For OBW, 5 factors were identified with lasso (insulin, CRP, fitness, adiponectin, and leptin), 6 with MARS (CRP, insulin, adiponectin, fitness, VEGF, and leptin), 3 with RF (CRP, insulin, and leptin), and 5 with BT (CRP, insulin, adiponectin, leptin, and fitness), of which, insulin, CRP, and leptin were selected by all four algorithms, while adiponectin and fitness by three algorithms (Table 1). As for OBWHO, 4 factors were identified with lasso (insulin, CRP, adiponectin, and fitness), 6 with MARS (CRP, insulin, adiponectin, sex, VEGF, and fitness), 3 with RF (CRP, insulin, and adiponectin), and 3 with BT (CRP, insulin, and adiponectin), of which CRP, insulin, and adiponectin were selected by all for algorithms (Table 1). Notably, leptin, a marker identified by all methods for OBW, was not chosen by any method for OBWHO. In terms of the classification performance of the models that generated the individual variable ranking, MARS (AUC = 0.837 and 0.853, respectively) was the top performer, while lasso was the least robust (AUC = 0.787 and 0.816, respectively).

**Figure 1:**
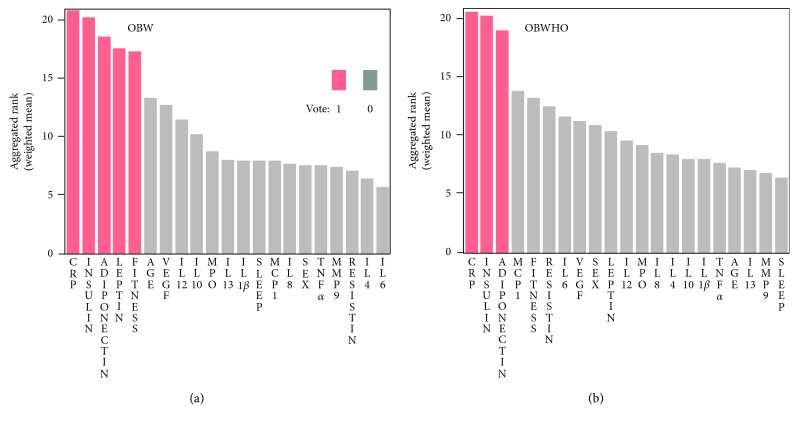
Aggregated ranking of all variables derived from variable importance rank lists generated from Lasso, MARS, RF, and BT. The variables in red indicate they were identified by at least three of the above algorithms (Vote as 1). For obesity as defined by (a) waist circumference (OBW) and (b) BMI (OBWHO).

Figure 1 illustrates the distribution of aggregated ranking of all variables, as calculated by averaging the rank ordering from all the rank lists, weighted by the classification performance of the models from which the individual variable ranking was derived. As shown, the top-ranking factors for OBW were CRP, insulin, adiponectin, followed by leptin and fitness, all of which were selected by a majority of the algorithms, as indicated in red. For OBWHO, on the other hand, the top ones were CRP, insulin, and adiponectin, selected by all algorithms. Leptin, a top feature for OBW, ranked 10th for OBWHO.

### 3.2. Subset of Top-Ranked Variables as Evaluated by Classification Performance

**Figure 2:**
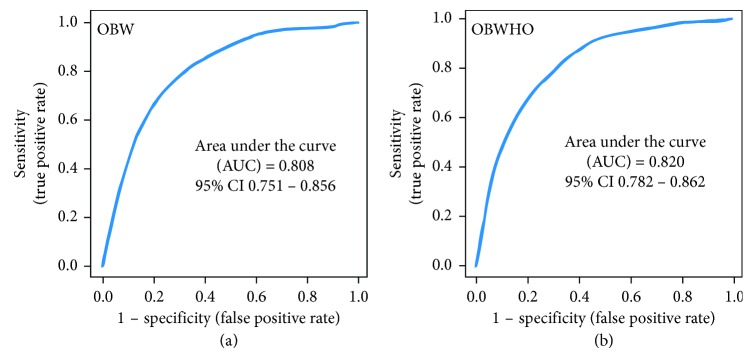
ROC analysis for classification performance using a subset of top-ranking variables from the aggregated rank list. The smooth ROC was averaged from 100 bootstrap samples, which provided the bootstrap percentile confidence interval. Support vector machine (SVM) was used as the classifier for obesity as defined by (a) waist circumference (OBW) and (b) BMI (OBWHO).

From the aggregated rank list, a subset of top-ranking variables that obtained a majority vote (i.e., identified by at least three algorithms) was used to evaluate their classification performance, with support vector machine (SVM) [8] as the classifier, using AUC from ROC analysis as the test metric. For OBW, the top 5 factors having a majority vote were tested (CRP, insulin, adiponectin, leptin, and fitness), achieving an AUC of 0.808 (95% CI: 0.751–0.856) (Figure 2(a)). For OBWHO, the top 3 factors having a majority vote were tested (CRP, insulin, and adiponectin), achieving an AUC of 0.82 (95% CI: 0.782–0.862) (Figure 2(b)).

### 3.3. Clustering of Obese Subjects Based on the Salivary Biomarkers and Clinical Measures

**Figure 3:**
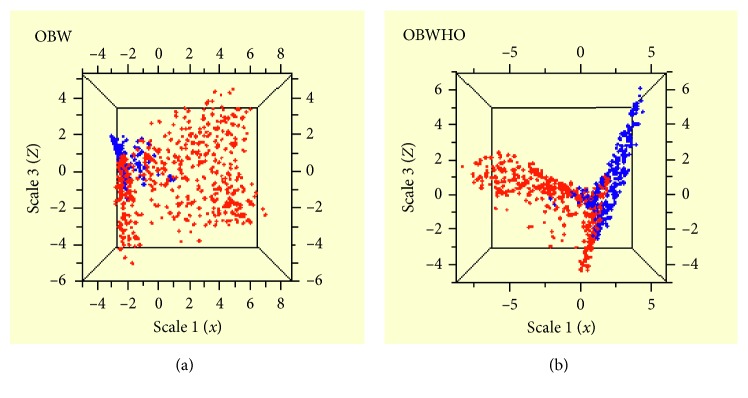
Tree-based clustering of subjects who were obese (in blue) versus those who were not obese (in red), for obesity as defined by (a) waist circumference (OBW) and (b) BMI (OBWHO).

The MDS plot generated from the tree-based proximity measures, based on biomarkers and other covariates, showed clustering of the obese subjects, for OBW as well as OBWHO (Figure 3). In Figure 3(a), obese subjects as defined by waist circumference (blue dots) were mostly clustered in the upper right corner, while the nonobese subjects were mostly dispersed everywhere, except for a small subset clustering on the left side. For OBWHO, however, the pattern was quite different (Figure 3(b)). Obese ones were clustered in a strip-like region to the right, while nonobese ones in a similar pattern to the left, with some parts of the two overlapping in the middle.

## 4. Discussion

Four data mining methods, logistic regression by lasso regularization (Lasso), multivariate adaptive regression spline (MARS), random forest (RF), and boosting classification trees (BT), identified diverse sets of salivary markers and other features associated with obesity, each generating a rank ordering of selected variables according to their relative importance. We used the ensemble idea for feature selection [16, 17] to construct an aggregated ranking aimed at obtaining a more robust subset, by averaging the ranking from individual algorithms, weighted by the classification performance of respective models that produced the ranking. As a result, CRP, insulin, adiponectin, leptin, and fitness emerged as the top-ranking factors identified with at least three algorithms for OBW, while CRP, insulin, and adiponectin were those for OBWHO. Finally, the above subset of variables was evaluated by their classification performance on phenotypes.

Instead of hypothesis-driven methods in search of associations involving parametric modeling and testing, we used data-driven approaches that are more flexible. They can handle large number of covariates which may be a limiting factor for conventional regression models and deal with other challenges from high-dimensional datasets such as extensive correlation among covariates, complex interactions among covariates, or nonlinear relationship between covariates and the response variable. For example, RF is well suited to capturing variables with strong interaction effect but minimum main effect, due to the increased probability for interactions to be detected in diverse trees, which would cause these interacting variables to be ranked higher in variable importance. MARS, on the other hand, is particularly suited to handling nonlinear associations, by using the linear spline to approximate nonlinear relationship. We explored an ensemble of four independent multivariate methods for variable selection, and it is reassuring to find they reached a consensus in placing certain salient variables on the top of the rank list, e.g., CRP, insulin, leptin for OBW, and CRP, insulin, and adiponectin for OBWHO. These salivary biomarkers correspond well to the established circulating plasma biomarkers associated with obesity [6], reflecting the metabolic aspect (e.g., insulin and leptin) as well as the inflammatory aspect (e.g., CRP and adiponectin) of the mechanism underlying obesity. However, no association was detected between obesity and salivary resistin, whose plasma counterpart has been known to correlate with obesity [6].

Variable selection is a process searching for a subset of best features. Depending on strategies used to retain relevant features, different learning algorithms may end up with feature subsets that are different local optima of the complete search space. Thus, by combining subsets from multiple methods, we might be able to expand the search space and yield a more robust feature subset to achieve better generalizability [16]. We adopted this ensemble idea for feature selection, creating a combined rank ordering by linear aggregation, in which the performance of the models generating individual ranking was allowed to influence the final ranking. There has been a recent development of another feature selection tool inspired by the ensemble idea, integrating eight feature selection methods [18]. Of note, this approach incorporates three univariate methods and five multivariate methods, of which four are variations from two different implementations of random forest algorithm. We believe that certain advantage could be gained if an ensemble approach includes distinctively different multivariate methods, as employed in our study.

It is interesting that we identified leptin as a top-ranking factor for obesity defined by waist circumference, but not for obesity defined by BMI. We know that waist circumference is more closely correlated with visceral fat [19], which is metabolically active where dysregulation of adipokine and cytokines acts as a key mechanism in obesity-related consequence, such as CVD [20–22]. Notably, salivary leptin has been found to exhibit a correlation coefficient of 0.78 with plasma level [23]. As leptin is a major adipokine, it follows that OBW subjects defined by abdominal fat have a higher average level of circulating leptin, which in turn leads to higher salivary leptin. OBWHO, on the other hand, reflects total body fat without regard to its distribution, thus resulting in lower average level of circulating leptin which leads to lower salivary leptin, dampening its correlation with the phenotype defined by BMI. Moreover, there are other issues that further confound the diagnosis of obesity measured by BMI, such as the cases of high muscularity, which complicates diagnosis of childhood obesity using BMI due to rapid body development [6]. Therefore, it is significant that salivary leptin is identified as the salivary biomarker mainly associated with OBW in children. As a side note, fitness, measured by heart rate elevation following a standard exercise [1], which has implications for long-term cardiovascular function, is also identified as a top-ranking factor for OBW.

The clustering pattern of obese subjects defined by these two measures is also quite different as revealed by tree-based clustering analysis. For OBW, the obese ones were close to one another, forming a relatively tight cluster (Figure 3(a)). For OBWHO, on the contrary, the obese ones dispersed across a much wider space (Figure 3(b)). This indicates that OBW subjects are relatively similar in terms of their profile of these salivary markers, whereas OBWHO subjects are much more varied. The clustering pattern from the OBWHO group indicates a greater heterogeneity of this population, with regard to the underlying mechanism of obesity, the distribution of fat tissues, and perhaps even the accuracy of obese status (e.g., highly muscular ones misclassified as obese).

This analysis using salivary samples contributes to biomarker studies in childhood obesity, which is not as abundant as those in adult population due to the difficulty of taking blood samples. Our findings suggest an importance of leptin in evaluating obesity by the waist circumference of children which does not appear when considering BMI percentile. Since leptin is an adipokine that is generally recognized as representing adipose tissue mass while BMI cannot differentiate between fat and lean muscle mass [6, 19], measurement by waist circumference is most relevant to obesity. This is particularly true for 10-year-old adolescents since their measurements were taken as puberty was being initiated. These observations are clearly consistent with the assumptions underlying the recommendation by the International Diabetes Foundation to use waist circumference to determine obesity of children [12].

## 5. Conclusion

There are a number of measures which have been used to evaluate obesity in children. In this study using salivary biomarkers, we have employed a data mining approach to discriminate between the two most common obesity measures via waist circumference and BMI. Although both were associated with salivary CRP, insulin, and adiponectin, leptin was unique for obesity evaluated by waist circumference. These results suggest that increased waist circumference is more closely related to adipocyte signaling that one would recognize as characterizing obesity and therefore a more sensitive measurement of obesity in children than BMI.

## Figures and Tables

**Table 1:** Important variables associated with OBW and OBWHO as identified by three algorithms.

Methods for the two obesity measures	Top-ranked variables^*∗*^ (in the order of relative importance)	AUC of respective classification models
*Obesity (OBW)*		
Lasso	Insulin, CRP, fitness, adiponectin, leptin	0.787
MARS	CRP, insulin, adiponectin, fitness, VEGF, leptin	0.837
Random forest	CRP, insulin, leptin	0.826
Boosting classification trees	CRP, insulin, adiponectin, leptin, fitness	0.816

*Obesity (OBWHO)*		
Lasso	Insulin, CRP, adiponectin, fitness	0.816
MARS	CRP, insulin, adiponectin, sex, VEGF, fitness	0.853
Random forest	CRP, insulin, adiponectin	0.833
Boosting classification trees	CRP, insulin, adiponectin	0.822

^*∗*^Variables with relative importance scores ≥ 20%.

## Data Availability

The data will be available upon request.

## Abbreviations

BMI: Body mass index
IL: Interleukin
Lasso: Least absolute shrinkage and selection operator
MARS: Multivariate adaptive regression spline
RF: Random forest.
